# Fatigue Analysis and Numerical Simulation of Loess Reinforced with Permeable Polyurethane Polymer Grouting

**DOI:** 10.3390/polym18020242

**Published:** 2026-01-16

**Authors:** Lisha Yue, Xiaodong Yang, Shuo Liu, Chengchao Guo, Zhihua Guo, Loukai Du, Lina Wang

**Affiliations:** 1 School of Water Conservancy and Transportation, Zhengzhou University, Zhengzhou 450001, China; 2 Hebi Engineering and Technology College, Henan Polytechnic University, Hebi Polytechnic, Hebi 458000, China; 3 School of Transportation Science and Engineering, Harbin Institute of Technology, Harbin 150000, China; 4 School of Civil Engineering, Sun Yat-sen University, Guangzhou 510275, China; 5 State Key Laboratory for Tunnel Engineering, Sun Yat-sen University, Guangzhou 510275, China; 6 Henan Huazhong Architectural Design Institute Co., Ltd., Zhengzhou 450001, China; 7 China Construction Fifth Engineering Division Corp., Ltd., Changsha 410000, China

**Keywords:** loess, polymer materials, grouting reinforcement, fatigue analysis, numerical simulation

## Abstract

Loess subgrades are prone to significant strength reduction and deformation under cyclic traffic loads and moisture ingress. Permeable polyurethane polymer grouting has emerged as a promising non-excavation technique for rapid subgrade reinforcement. This study systematically investigated the fatigue behavior of polymer-grouted loess using laboratory fatigue tests and numerical simulations. A series of stress-controlled cyclic tests were conducted on grouted loess specimens under varying moisture contents and stress levels, revealing that fatigue life decreased with increasing moisture and stress levels, with a maximum life of 200,000 cycles achieved under optimal conditions. The failure process was categorized into three distinct stages, culminating in a “multiple-crack” mode, indicating improved stress distribution and ductility. Statistical analysis confirmed that fatigue life followed a two-parameter Weibull distribution, enabling the development of a probabilistic fatigue life prediction model. Furthermore, a 3D finite element model of the road structure was established in Abaqus and integrated with Fe-safe for fatigue life assessment. The results demonstrated that polymer grouting reduced subgrade stress by nearly one order of magnitude and increased fatigue life by approximately tenfold. The consistency between the simulation outcomes and experimentally derived fatigue equations underscores the reliability of the proposed numerical approach. This research provides a theoretical and practical foundation for the fatigue-resistant design and maintenance of loess subgrades reinforced with permeable polyurethane polymer grouting, contributing to the development of sustainable infrastructure in loess-rich regions.

## 1. Introduction

Loess is a yellow, silty sedimentary deposit formed by Quaternary aeolian processes, primarily distributed in mid-latitude regions of both hemispheres [1,2]. It covers a vast area, accounting for approximately 9.3% of the world’s land surface, with China being the primary country in Asia where loess is distributed [3,4,5]. Loess is a geological formation developed under arid–semi-arid conditions, containing abundant silt particles and carbonates. It exhibits distinctive physical and mechanical properties: a metastable structure, high porosity, and water sensitivity. In its natural state, loess exhibits considerable strength and is resistant to compression. However, upon wetting, soluble salts rapidly dissolve, causing a swift reduction in strength and significant volumetric deformation. This deformation process is characterized by abruptness, discontinuity, and irreversibility [6,7,8]. Loess’s characteristics mean that roads constructed in loess regions, when subjected to adverse natural weather conditions, may experience water infiltration into the subgrade due to defects such as cracks in the upper structural layers of the road. This leads to the formation of wet, soft structural layers [9,10,11,12,13]. Simultaneously, the action of external road loads causes the bearing capacity of loess subgrades to sharply deteriorate under water–load coupling, leading to subgrade instability and thereby compromising the stability of the entire road structure. Particularly when subjected to rainwater erosion, the weakened bearing capacity renders the subgrade highly susceptible to severe defects, such as cavities and subsidence [14,15,16]. These phenomena damage highway infrastructure, directly or indirectly causing substantial economic losses while severely constraining local economic development and construction. Consequently, enhancing the bearing capacity of existing loess subgrades requires urgent resolution.

**Figure 1:**
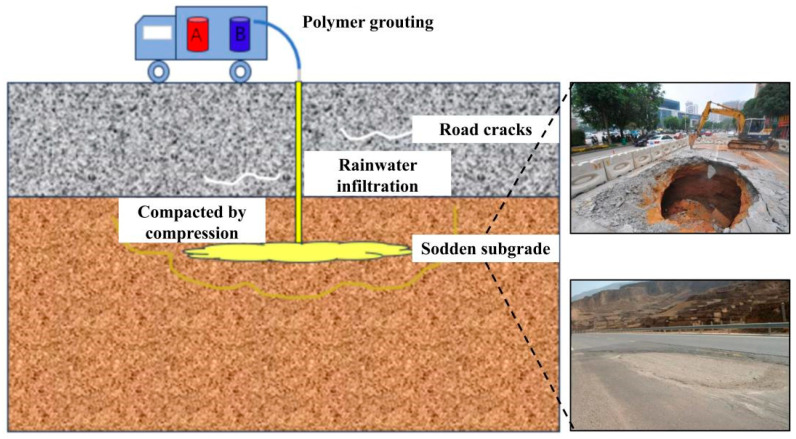
Schematic diagram of grouting reinforcement for subgrade.

The primary methods employed to enhance the load-bearing capacity of loess subgrade defects include compaction piling, grouting reinforcement, and excavation and replacement of defective subgrade sections using chemical materials [17,18,19]. Choobbasti et al. [20] reinforced loess with nano-calcium carbonate carpet waste fibers. Their experiments revealed that nano-calcium carbonate enhances the mechanical properties of loess, improves micro-fractures within the soil, and binds soil particles into a cohesive structure. Jia et al. [21] investigated the use of lime to reinforce loess. They observed that the hydration reactions occurring during the solidification process removed excess moisture from the soil, thereby increasing its compaction and enhancing its mechanical properties. Gu et al. [22] investigated the reinforcement effect of calcium carbonate on loess at both macro- and micro-levels, finding that the macro- and micro-properties of reinforced loess were significantly improved. Tabarsa et al. [23] employed nano-clays to enhance the mechanical properties of loess, observing in field trials that a higher nano-clay content correlated with reduced erosion susceptibility and improved scour resistance. Kong et al. [24] discovered that nano-silica significantly enhances the mechanical properties of loess and investigated the mechanism of nano-silica reinforcement at the microscopic level. Both compaction pile and chemical material reinforcement require excavation of the roadbed, with extensive excavation operations producing unnecessary resource wastage. Furthermore, conventional grouting materials necessitate a curing period after application, preventing rapid traffic resumption. Polymer grouting technology constitutes a non-excavation, rapid remediation technique for concealed subgrade defects. permeable polyurethane polymer grouting materials are injected into weakened subgrade zones via grouting pipes, utilizing an integrated, multi-functional polymer grouting system [25,26,27,28]. It leverages the permeation and bonding properties of polymer grout to achieve rapid filling of subgrade weak zones and soil compaction via cohesive bonding, thereby enhancing the subgrade bearing capacity [29,30]. Compared with conventional road defect remediation techniques, using polymer grouting for subgrade reinforcement enables rapid reopening to traffic. From Figure 1, this method employs drilling for injection repairs, which significantly reduces damage to the original road structure and avoids the resource wastage associated with excavation, thereby aligning with green ecological construction principles.

Fatigue behavior constitutes a crucial aspect in the study of soil properties. Several scholars have researched the fatigue characteristics and patterns of soil. Li et al. [31] conducted experimental analyses of the mechanical and fatigue properties of foamed lightweight soil, deriving a fatigue equation for this material. They analyzed the service life of foamed lightweight soil used as a construction material for highway subgrades based on the stress–strain characteristics of actual pavement structures. There are three primary categories of fatigue characterization models for soils: phenomenological models based on fatigue testing analysis (S-N fatigue equation characterization methods), fatigue crack propagation models based on fracture mechanics, and fatigue damage evolution models based on damage mechanics. Zhao et al. [32] established a cohesive damage model to characterize the fatigue damage behavior of cement-stabilized soil, describing its fatigue decay characteristics under cyclic loading. Zhang et al. [33] introduced a cohesive force model to investigate the fatigue cracking behavior of stabilized soil, employing finite element analysis software to examine the fatigue damage behavior of complex materials, such as stabilized soil. Lv et al. [34] found that the loading rate significantly influences the strength of cement-stabilized soil, establishing a fatigue damage model for such materials. Existing research on soil fatigue behavior has predominantly focused on cement-stabilized soils and concrete. Studies investigating the performance changes and fatigue failure mechanisms of loess, particularly stabilized loess, under repeated loading conditions remain insufficient. Given that loess subgrades frequently endure cyclic traffic loads during operation, conducting fatigue tests and employing numerical simulation software to predict their fatigue life is essential [35].

Despite the promising advancements in polymer grouting technology for soil improvement, a conspicuous research gap persists in the systematic evaluation of its long-term fatigue performance under cyclic loading, particularly for moisture-sensitive loess subgrades. Existing studies have predominantly focused on the short-term mechanical enhancement of polymer-grouted soils, such as increased compressive strength, improved stiffness, and reduced permeability. However, the durability and fatigue resistance of these composite materials under repeated traffic remain poorly understood. Current fatigue models for stabilized soils largely derive from cementitious or chemically treated materials, whose failure mechanisms and crack propagation behaviors differ fundamentally from those of polymer-reinforced loess. Moreover, while numerical simulations have been employed to predict the fatigue life of conventional pavement materials, the integration of reliable constitutive models for polymer-grouted loess is notably absent. Therefore, there is a critical need to investigate the fatigue behavior of permeable polyurethane polymer-grouted loess through integrated experimental and numerical approaches, addressing the combined effects of cyclic stress amplitude, moisture variation, and composite material degradation over time.

This study primarily employed permeable polyurethane polymer grouting to reinforce loess subgrades, thereby enhancing their load-bearing capacity. It systematically investigated their fatigue performance and established a road model to validate the results. This study aimed to address the inadequate load-bearing capacity of existing loess subgrades and provide theoretical support for the operation and maintenance of similar roads, offering practical reference and guidance for field applications.

## 2. Materials and Methods

In practical engineering applications, loess subgrades reinforced with permeable polyurethane polymer grouting are frequently subjected to cyclic traffic loads. Consequently, conducting fatigue tests to simulate the repetitive loading experienced by grout-reinforced loess subgrades is essential. This approach reveals the degradation patterns of loess subgrades under repeated stress cycles, thereby enabling predictions of their service life and safety. This study investigated the performance degradation patterns of permeable polyurethane polymer grout-reinforced loess subgrades under repeated loading using fatigue testing, thereby elucidating their fatigue failure mechanisms.

### 2.1. Experimental Materials

#### 2.1.1. Characteristics of the Loess

**Figure 2:**
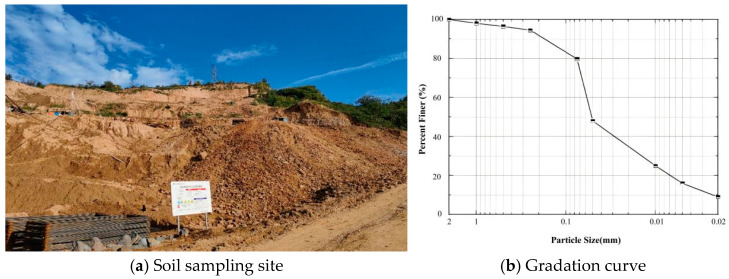
Loess and gradation.

The loess used in the tests was sourced from a motorway, exhibiting a yellowish-brown color. The sampling site is depicted in Figure 2a, with soil extracted from depths ranging from 0 to 3 m. Following the Standard Specification for Geotechnical Test Methods (GB/T 50123-2019) [36], the collected loess underwent particle size analysis, compaction tests, specific gravity tests, and liquid and plastic limit tests. The collected loess was subjected to particle size analysis, with particles larger than 0.075 mm being sieved using the sieving method [37]. A 500 g soil sample was dried, sieved, and weighed after vibratory sieving. The weight of soil particles retained on each sieve mesh was recorded, and the percentage distribution of particles in each size fraction was calculated accordingly. The specific sieve analysis results are shown in Table 1, with the particle size distribution curve depicted in Figure 2b. Compaction tests were conducted on the loess used in this experiment, establishing a maximum dry density of 1.76 g/cm^3^ and an optimum moisture content of 12.7%.

The fundamental physical properties of loess were determined using specific gravity tests and liquid and plastic limits tests, as shown in Table 2.

Moisture content is the primary factor influencing compressive strength; consequently, this study’s compressive fatigue tests primarily investigated the fatigue life of grouted specimens under varying moisture contents and stress levels [38,39]. The most favorable combinations of compaction degree and curing duration were selected for each moisture content. Consequently, the specimen moisture contents were set at 13%, 16%, and 19%, the compaction degree at 70%, the curing duration at 28 days, and the stress levels at 0.6, 0.7, 0.8, and 0.9.

#### 2.1.2. Polymer Grouting Material

**Figure 3:**
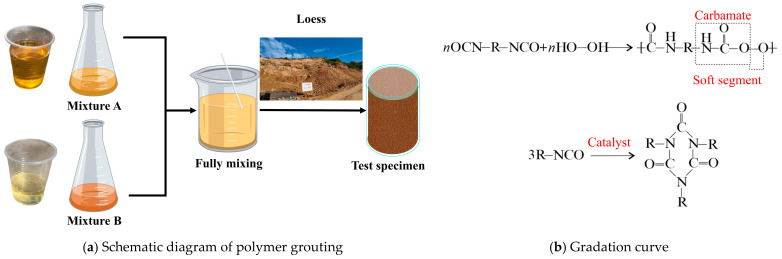
Polymer slurry.

The grouting material employed was a highly permeable formulation independently developed by the research team [40]. This material comprises two components, designated as Component A and Component B, which must be uniformly mixed in a 1:1 mass ratio prior to application. Component A primarily consists of polyether polyols, surfactants, and dimethylformamide, presenting as a deep yellow liquid. Component B is primarily composed of poly (methylene polyphenyl) polyisocyanate and toluene diisocyanate, appearing as a pale-yellow liquid. The visual characteristics of both components are illustrated in Figure 3a, with their fundamental physical properties summarized in Table 3 [41,42]. Table 4 shows the molecular weight of polymer materials. This permeable polyurethane polymer grouting material exhibits high permeability, low viscosity, high bonding strength, and excellent durability. When subjected to pressure, the material effectively fills soil particle voids, significantly enhancing the mechanical properties of the soil mass. Figure 3b shows gel reaction and trimrization reaction of isocyanurate. The strength-generating mechanism is the polyurethane polymerization reaction between the hydroxyl (-OH) groups of the polyols (Component A) and the isocyanate (-NCO) groups of Component B, forming urethane linkages. This reaction is exothermic and proceeds rapidly upon mixing, leading to the formation of a three-dimensional polymer network. Concurrently, a portion of the isocyanate groups can undergo a trimerization reaction, catalyzed by conditions within the soil mass, to form stable isocyanurate rings. As illustrated in Figure 3b, these isocyanurate structures contribute to enhanced thermal stability and rigidity in the cured polymer. The resulting solid is a crosslinked polymer elastomer with a cellular structure, whose final mechanical properties are a direct consequence of this chemical formulation and curing process.

It is noted that Component A contains dimethylformamide (DMF) as a solvent and processing aid. DMF exhibits moderate volatility (boiling point ~153 °C) and is largely expelled from the system during the exothermic polymerization and curing process. Under typical in situ conditions, the porous structure of loess facilitates the diffusion and evaporation of residual DMF, minimizing its long-term retention in the soil matrix. Prior studies on similar polyurethane grouts have shown that the rapid gelation and hydrophobic nature of the cured polymer limit any potential interaction between organic solvents and soil minerals, ensuring the environmental compatibility and mechanical stability of the grouted composite.

### 2.2. Test Procedure

#### 2.2.1. Test Apparatus

**Figure 4:**
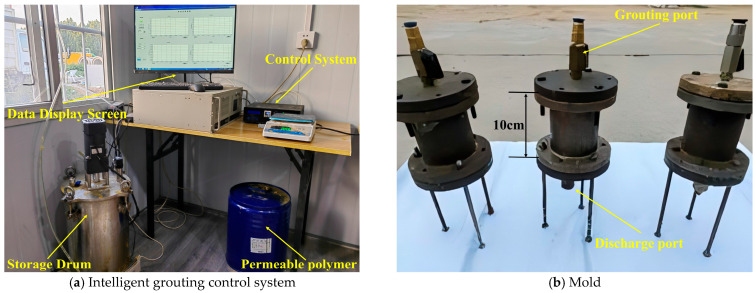
Grouting equipment and forming molds.

As illustrated in Figure 4a, the indoor grouting equipment comprises a constant-pressure grouting system independently designed by the research team. It primarily consists of an air compressor, an intelligent pressure control system, a material storage tank, and a soil-loading mold. The system can adjust the pressure during grouting in real time and monitor the grout flow rate, thereby ensuring the stability and controllability of the grouting process. The soil mold possesses adequate strength and durability. The inner and outer diameters of the upper and lower cover plates are 50 mm and 90 mm, respectively, and the mold cavity height is 100 mm. The soil mold is illustrated in Figure 4b. Based on the dimensions of the grouting mold, the specimen obtained after grouting was a cylindrical body with a diameter of 50 mm and a height of 100 mm. This grouting system is simple to operate and enables constant-pressure grouting, ensuring the stability of the grouting process. It is suitable for grouting and reinforcing loess specimens in laboratory settings, providing reliable foundational conditions for subsequent experimental research.

**Figure 5:**
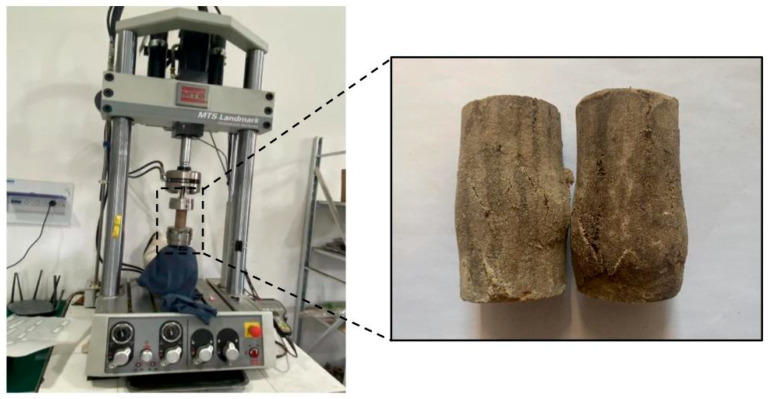
Axial fatigue testing system.

The fatigue testing apparatus employed a 15 kN axial fatigue testing system, as illustrated in Figure 5. This system permits loading frequencies ranging from 0 to 10 Hz, with a load capacity of ±250 kN and a sampling frequency of 122 kHz. It features sine, square, triangular, harmonic, hold, load spectrum, and custom waveform capabilities, enabling mechanical property testing of materials and structural components, including static testing, low-cycle fatigue testing, high-cycle fatigue testing, three-point bending fatigue testing, and fracture testing. The fatigue specimens were circular soil columns measuring 50 mm in diameter and 100 mm in height.

#### 2.2.2. Setting of Test Parameters

When conducting fatigue tests on subgrade materials, selecting appropriate loading frequencies, loading waveforms, stress levels, and cyclic characteristics is crucial for simulating actual service conditions and evaluating material performance.

The loading frequency was set at 10 Hz, corresponding to a vehicle traveling at 80 km/h, which aligns with the design speed specified in the Technical Standards for Highway Engineering (JTG B01-2014) [43]. Consequently, establishing 10 Hz as the loading frequency for the test better simulated actual road operational conditions. The loading waveform significantly influences material fatigue life, with the stress–strain effects induced by vehicular loads on roads resembling a sinusoidal curve. Employing a sinusoidal waveform as the loading pattern more accurately simulates the actual load conditions experienced by roadbeds; hence, this trial selected a sinusoidal waveform for loading to achieve more precise fatigue life predictions. The stress level, defined as the ratio of repeated stress to ultimate stress (compressive strength), directly influences the fatigue life of specimens. Selecting an appropriate stress level ensures the test’s validity and practicability. If the stress level is too low, the test cycle will be excessively prolonged. If the stress level is too high, it may cause premature failure of the specimen, thereby underestimating the material’s actual fatigue performance. Zero-point drift may occur during continuous cyclic loading tests, causing separation between the loading end and the specimen surface, which can induce impact loading. A cyclic characteristic value of 0.1 was set during testing to prevent this, representing the ratio of the minimum to maximum loading forces. This ensured test stability and reliability, thereby guaranteeing the accuracy of the test data.

This compressive fatigue testing primarily investigated the fatigue life of grouted specimens under varying moisture contents and stress levels. For each moisture content, the most favorable combination of compaction degree and curing duration was selected. Consequently, the specimen moisture contents were set at 13%, 16%, and 19%, the compaction degree at 70%, the curing duration at 28 days, and the stress levels at 0.6, 0.7, 0.8, and 0.9. The peak load stresses applied during fatigue testing for each moisture content are detailed in Table 5.

## 3. Result Analysis

### 3.1. Analysis of Fatigue Loading Results

The fatigue loading results for the specimens at different moisture contents and stress levels are presented in Table 6, showing that the specimens’ fatigue life data exhibited a certain degree of dispersion. This may be attributable to multiple factors, such as the non-uniform distribution of polymer slurry within the specimens and irregular specimen morphology. The specimens demonstrated extreme sensitivity to applied stress levels, and combined with inherent measurement system errors, this contributed to the dispersion observed in the fatigue life data. Four parallel test sets were conducted for each operating condition to reduce data dispersion and obtain more reliable results. The fatigue test data indicate that the specimens could achieve fatigue lifetimes of up to 200,000 cycles. The injection of high polymers effectively introduced micro-springs within the specimens. These ‘spring elements’ enhanced the specimens’ fatigue resistance, enabling a better distribution of cyclic loads and delaying fatigue failure. The specimens’ fatigue life is related to stress levels and moisture content. The load borne by specimens gradually approaches its bearing limit as stress levels increase, reducing fatigue life. This demonstrates the specimens’ sensitivity to fatigue under high-stress conditions. The specimens’ fatigue life diminishes with increasing moisture content at identical stress levels. Fewer internal voids exist within the specimen at lower moisture levels, resulting in more pronounced cementation between soil particles. This enhances the frictional resistance between the polymer and soil particles, thereby strengthening the specimen’s capacity to withstand external loads. Consequently, permeable polymer grouting reinforcement of loess enhances its fatigue life.

In compression fatigue testing, loess specimens reinforced with grouting exhibited failure modes highly similar to those observed in static compression tests under fatigue loading, with fine cracks appearing before failure. These minute cracks progressively propagated under sustained cyclic loading, ultimately leading to specimen failure. The fatigue failure process can be divided into three distinct stages based on their percentage contribution to the total fatigue life cycle:

(1) The initial stage accounts for approximately 10% to 15% of the total fatigue life. Microcracks begin to form within the specimen, and fatigue strain starts to increase. During this phase, specimens contain a small number of pores and fissures. As the number of cycles increases, the pores between particles gradually diminish under cyclic loading, and fissures progressively close. Components such as loess particles and polymers within the specimen become denser under cyclic loading. No damage occurs internally, yet fatigue strain continues to increase.
(2) The mid-stage accounts for approximately 70–80% of the total fatigue life. Microcracks grow steadily, with microscopic cracks developing into macroscopic cracks, while the fatigue strain remains unchanged. Concurrently, minute fissures emerge within the specimen, with both their quantity and width increasing with accumulated cycles. Under cyclic loading, deformation and displacement occur between the soil particle skeleton and polymer matrix, disrupting the adhesive bond between the soil particles and polymer. This facilitates the propagation of existing internal fissures while generating new cracks extending outward.
(3) During the final stage, the total strain increases sharply, leading to specimen failure within a relatively short period. This phase accounts for 10% to 15% of the fatigue life. Fatigue strain increases rapidly during this stage; at this point, the soil stress reaches its ultimate value; and existing microcracks within the specimen propagate further, with all fissures interconnecting to form a fracture surface, all of which result in specimen failure.

**Figure 6:**
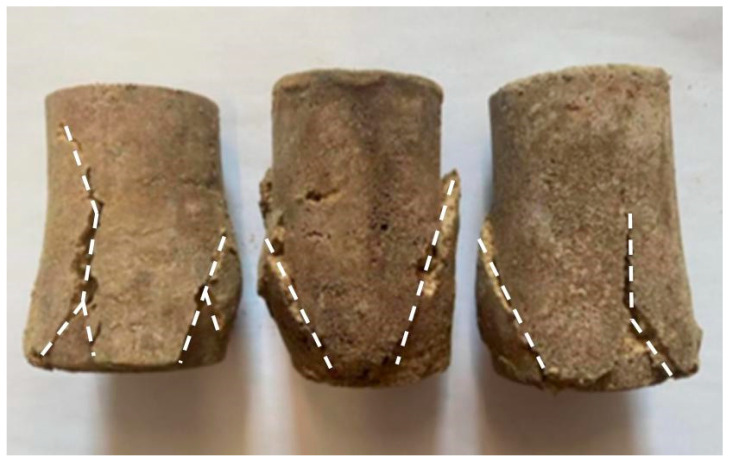
Fatigue failure mode of the test specimens.

The interconnected cracks caused bulging in the specimen, ultimately resulting in a ‘multiple-crack’ failure mode, as illustrated in Figure 6. This ‘multiple-crack’ failure pattern demonstrates that polymer grout-reinforced specimens can more uniformly distribute stresses during fatigue loading. This mitigates the effects of localized stress concentration, enhances the specimen’s deformation capacity, and renders ductile failure characteristics particularly pronounced. As a resilient material, the permeable polyurethane polymer functions akin to distributing countless micro-springs throughout the loess specimen. The injection of the permeable polyurethane polymer creates a coherent, elastic matrix that permeates the soil pores and bonds the particles. This three-dimensional polymer network enhances the specimen’s fatigue resistance by effectively redistributing cyclic loads through its elastic deformation and by dissipating energy at the polymer-soil interfaces. This mechanism delays the initiation and coalescence of fatigue-induced microcrack.

**Figure 7:**
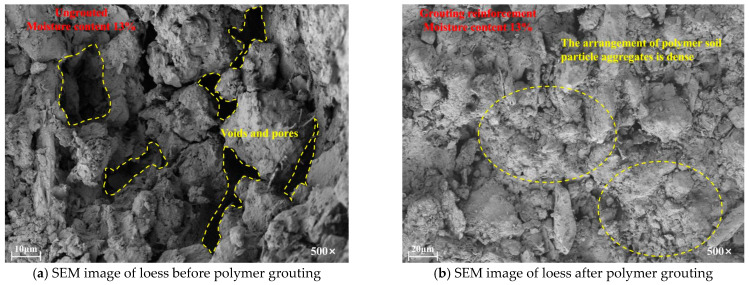
SEM images of loess before and after polymer grouting.

Under scanning electron microscopy at 500× magnification, the ungrouted loess specimen still exhibits numerous voids and large pores internally. As shown in Figure 7, the polymer successfully fills the pores within the loess, effectively reducing the pore volume. This is also a key factor in enhancing the specimen’s overall strength and fatigue resistance.

### 3.2. Establishment of Fatigue Life Equations

#### 3.2.1. Fatigue Life Prediction Model

An appropriate fatigue life estimation model must be selected to predict the fatigue life of loess subgrades after permeable polyurethane polymer grouting. A fatigue equation for loess specimens after permeable polyurethane polymer grouting should be established, with the stress levels experienced by the subgrade during actual operation subsequently substituted into this fatigue equation to forecast the fatigue life of the grouted loess subgrade. Fatigue performance may be characterized by S-N curves and ε-N curves. The fatigue testing of polymer grout-reinforced loess specimens employed a stress control methodology; hence, S-N curves were employed to depict the relationship between the stress level S and the fatigue life N. S-N curves primarily adopt exponential or power law forms, also termed single-logarithmic and double-logarithmic formats [44].

The numerical function type (single logarithmic function type) is expressed as

(1) $$Ne^{as}=c.$$

Taking the logarithm of both sides of Equation (1) yields the following.

Transforming into Equation (2), we obtain

(2) S=a−lgN.

In Equation (2), S denotes the stress level, N represents the fatigue life, and a, b, and c are specific parameters of the tested material. As shown in Equation (2), the curve exhibits a clear linear relationship in a single-logarithmic coordinate system, allowing parameters a and b to be determined using linear regression methods.

The power function type (double-logarithmic function type) is expressed as

(3) $$S^{\alpha }N=c.$$

Taking the logarithm of both sides of the above equation yields the following.

Transforming into Equation (4), we obtain

(4) lgS=a−blgN.

In Equation (4), S denotes the stress level, N represents the fatigue life, and a, b, and c are parameters specific to the tested material.

Following statistical principles, test data may be used to establish S-N curves based on different guarantee rates, P, forming P-S-N curves, to ensure the accuracy of extensive fatigue life data. The fatigue performance model for grouted loess specimens was established using Equations (2) and (4).

#### 3.2.2. Probability Model of Fatigue Life Distribution

As shown in Table 5, the fatigue life of the loess specimens after grouting exhibited a certain degree of dispersion. Probabilistic statistics and fatigue reliability principles were employed to conduct a rational investigation into the fatigue life of loess specimens to accurately reflect the variation patterns of fatigue life under different stress levels and moisture contents. Typically, the Weibull distribution, log-normal distribution, and normal distribution are utilized for analyzing and processing materials’ fatigue life.

(1) Normal distribution

A normal distribution is also known as a Gaussian distribution. In practical engineering applications, a normal distribution is the most commonly encountered distribution function. The probability density function for a normal distribution is expressed as

(5) $$f(N)=\frac{1}{\sqrt{2\Pi }\sigma }e^{-\frac{\left(N-\mu \right)}{2\sigma^{2}}}(-\infty <N<+\infty ).$$

In the formula, N denotes the fatigue life, and μ and σ represent the mean and variance of N, respectively.

The above equation shows that a normal distribution exhibits a significant drawback: when the random variable N < 0, its probability density function $P(N<0)=\int_{-\infty }^{0}\frac{1}{\sqrt{2\pi }\sigma }e^{\frac{{\left(N-\mu \right)}^{2}}{2\sigma^{2}}}dx>0$ deviates from the actual circumstances. Consequently, a normal distribution is unsuitable for modeling the fatigue life distribution of loess specimens following polymer grouting.

(2) Log-normal distribution

A log-normal distribution is based on a normal distribution. Typically, the random variable N from a normal distribution undergoes a logarithmic transformation into lgN, forming the random variable of the log-normal distribution. Its probability density function is expressed as

(6) $$f(lgN)=\frac{1}{\sqrt{2\pi \sigma_{lnN}}}exp\left[\frac{{\left(lnN-\mu_{lnN}\right)}^{2}}{2\sigma^{2}lnN}\right](-\infty <lnN<+\infty ).$$

In the formula, lgN denotes fatigue life, while μlnN and σlnN represent the mean and variance of lnN, respectively.

The above equation shows that applying a logarithmic transformation to the normally distributed random variable N (lgN) maps values from the real number interval (0, +∞) to the entire real number interval (−∞, +∞). This circumvents the shortcomings of a normal distribution, aligning more closely with practical scenarios. However, a log-normal distribution also possesses drawbacks. When fatigue life N = 0, the distribution function F(N) > 0; that is, a small number of specimens fail before undergoing fatigue testing. Consequently, employing a log-normal distribution to process fatigue life yields overly conservative results.

(3) Weibull distribution

The Weibull distribution, also known as the Weber distribution, is a probability density function proposed by the Swedish scientist Weibull to describe materials’ fatigue life. It generally takes two forms: the three-parameter and two-parameter Weibull distributions. The probability density function for the former is expressed as

(7) $$f(N)=\frac{b}{N_{a}-N_{0}}{\left(\frac{N-N_{0}}{N_{a}-N_{0}}\right)}^{b-1}exp\left[-{\left(\frac{N-N_{0}}{N_{a}-N_{0}}\right)}^{b}\right](N_{0}<N<\infty ).$$

In the equation, N denotes fatigue life, represents the characteristic life parameter, and denotes the minimum life parameter, and b is the Weibull shape parameter. Considering the significant variability in fatigue life when reinforcing loess with polymer grouting, the minimum life parameter is typically set to 0 for computational convenience and more reliable experimental results. When it is 0, a two-parameter Weibull distribution is obtained, whose probability density function is expressed as

(8) $$f(N)=\frac{b}{N_{a}}{\left(\frac{N}{N_{a}}\right)}^{b-1}exp\left[-{\left(\frac{N}{N_{a}}\right)}^{b}\right]\;(0<N<\infty ).$$

In the formula, N denotes fatigue life, Na denotes the characteristic life parameter, and b denotes the Weibull shape parameter.

Equations (7) and (8) show that the Weibull distribution possesses a minimum safe life compared with the other two distributions. Moreover, the safe life still aligns with the actual fatigue failure behavior within a higher reliability range. Therefore, in practical engineering applications, employing the Weibull distribution to analyze material fatigue life offers greater applicability and reliability.

In summary, having reviewed three commonly employed probability models for fatigue life distribution, this study decided to apply the Weibull distribution to analyze the fatigue life of loess specimens reinforced with polymer grouting. This approach yielded the corresponding curve and established the requisite fatigue equation.

#### 3.2.3. Weibull Distribution Test of Fatigue Life

A two-parameter Weibull distribution was employed for preliminary summarization to analyze the fatigue life of loess specimens reinforced with polymer grouting. Firstly, it was necessary to verify whether the fatigue life of these specimens conformed to a two-parameter Weibull distribution. If so, mathematical statistical methods could be applied to plot curves based on different assurance levels and moisture contents, thereby establishing a fatigue equation grounded in the Weibull distribution.

Based on the two-parameter Weibull distribution probability density function f(N) in Equation (8), the calculation formula for the Weibull distribution function F(Np) can be derived:

(9) $$F\left(N_{p}\right)=P\left(N_{\xi }<N_{p}\right)=\int_{N_{\xi }}^{N_{p}}f\left(N\right)dN=\int_{N_{\xi }}^{N_{p}}\frac{b}{N_{a}}{\left(\frac{N}{N_{a}}\right)}^{b-1}exp\left[-{\left(\frac{N}{N_{a}}\right)}^{b}\right]dN.$$

Integrating Equation (9) yields

(10) $$F\left(N_{p}\right)=P\left(N_{\xi }<N_{p}\right)=1-exp\left[-{\left(\frac{N}{N_{a}}\right)}^{b}\right].$$

The Weibull distribution function F(Np) represents the failure probability of a specimen. Therefore, the reliability P of the specimen can be expressed as

(11) $$P=1-F\left(N_{p}\right)=exp\left[-{\left(\frac{N}{N_{a}}\right)}^{b}\right].$$

This is followed by taking the derivative of Equation (11), applying double-logarithmic transformation to both sides, and then rearranging by changing the base of the logarithm yields

(12) $$-\ln\ln\left(\frac{1}{p}\right)=-2.303b\mathrm{lg}N_{p}+2.303b\mathrm{lg}N_{a}.$$

In Equation (12), P denotes the reliability of the specimen, Np represents the fatigue life at the guarantee rate P, Na is the characteristic life parameter, and b represents the Weibull shape parameter.

According to Equation (12), under the two-parameter Weibull distribution, a clear linear relationship exists between −ln ln(1/P) and the logarithm of fatigue life, lgNp. This equation may be employed to verify whether experimental data conform to a two-parameter Weibull distribution. By performing regression analysis on fatigue life data, if a good linear relationship is observed between −ln ln(1/P) and ln Np, it may be concluded that the fatigue life data satisfy the two-parameter Weibull distribution. The consistency between the fatigue life of grouted loess specimens and the two-parameter Weibull distribution was verified using graphical methods. The specific verification procedure was as follows.

**Figure 8:**
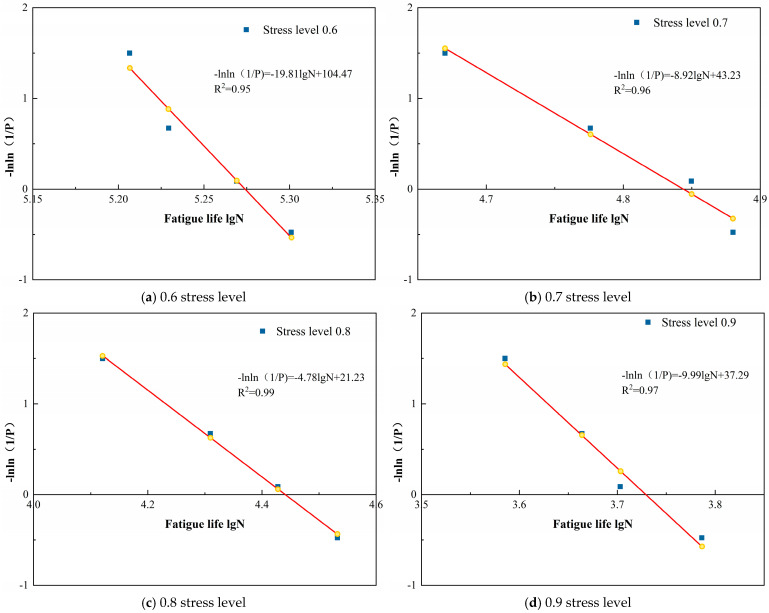
Weibull distribution verification of fatigue life at different stress levels for 13% moisture content.

**Figure 9:**
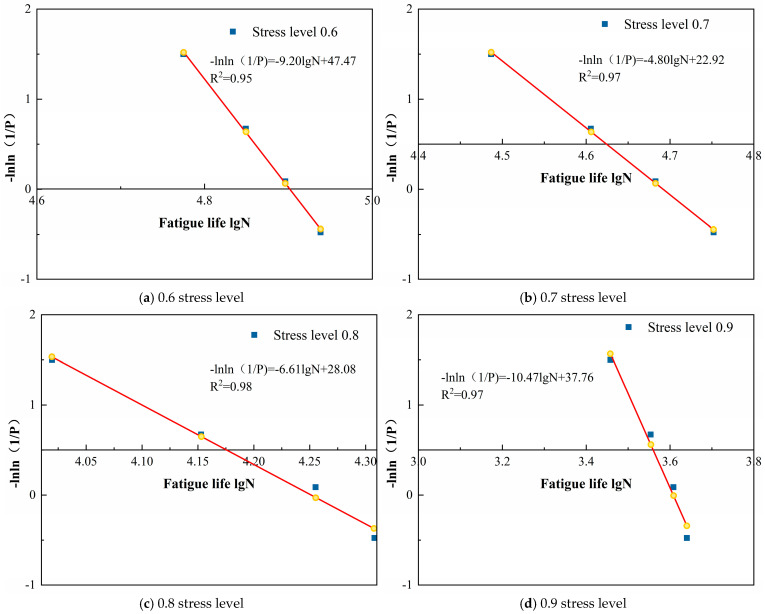
Weibull distribution verification of fatigue life at different stress levels for 16% moisture content.

**Figure 10:**
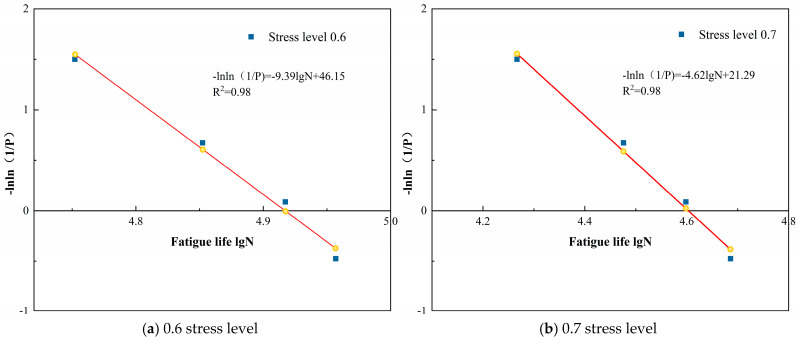

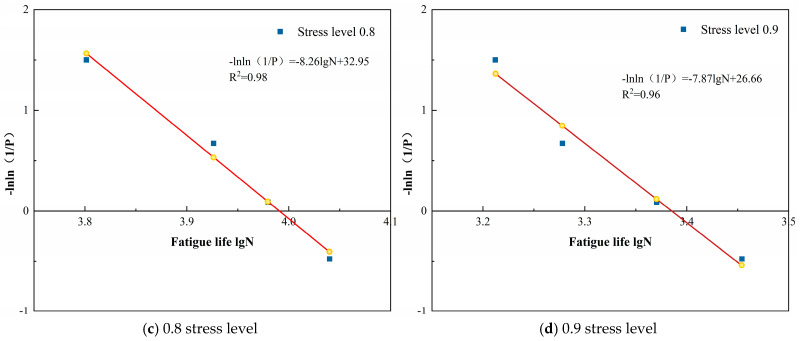
Weibull distribution verification of fatigue life at different stress levels for 19% moisture content.

Based on the aforementioned content, a two-parameter Weibull distribution analysis was conducted on the fatigue life of loess specimens reinforced with polymer grouting under varying stress levels and moisture content conditions, as presented in Table 6. The results are shown in Table 7 and Figure 8, Figure 9 and Figure 10.

Figure 8, Figure 9 and Figure 10 demonstrate that the linear regression analyses conducted on logarithmic fatigue life versus −ln ln(1/P) under varying stress levels and moisture content conditions yielded correlation coefficients exceeding 0.94. This indicates that a significant linear relationship existed between logarithmic fatigue life and −ln ln(1/P) across varying stress levels, confirming that the fatigue life of loess specimens reinforced with polymer grouting substantially followed a two-parameter Weibull distribution. Consequently, the two-parameter Weibull distribution is applicable for analyzing the fatigue life of grout-reinforced loess specimens.

#### 3.2.4. Fatigue Equation Based on Weibull Distribution

The fatigue life of polymer-grouted specimens was well-described using a two-parameter Weibull distribution. Consequently, based on the fitting equations presented in Figure 8, Figure 9 and Figure 10, the logarithmic fatigue life could be determined for different stress levels and arbitrary reliability levels. This paper focused solely on reliability levels of 50% and 95%. The logarithmic fatigue life under varying stress levels and moisture contents is summarized in Table 8.

**Figure 11:**
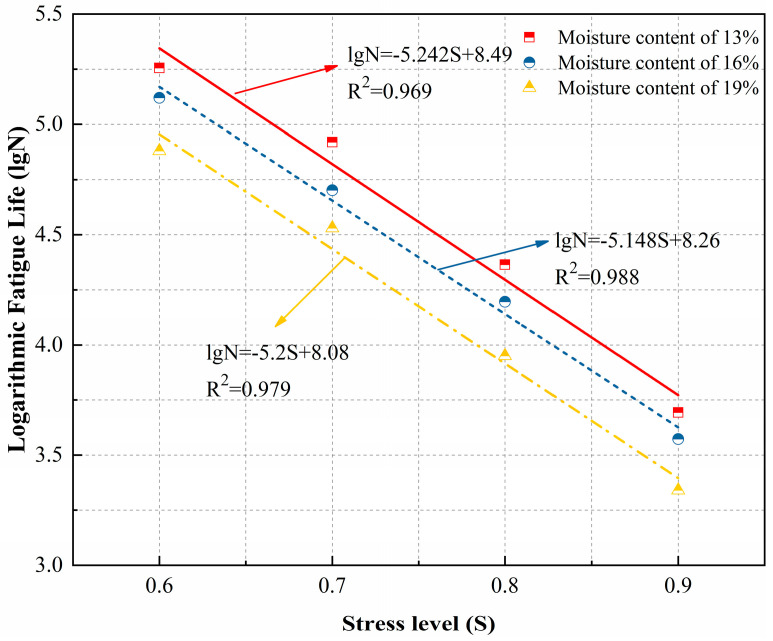
P-S-lgN curve of the Weibull distribution at a 0.50 guarantee rate.

**Figure 12:**
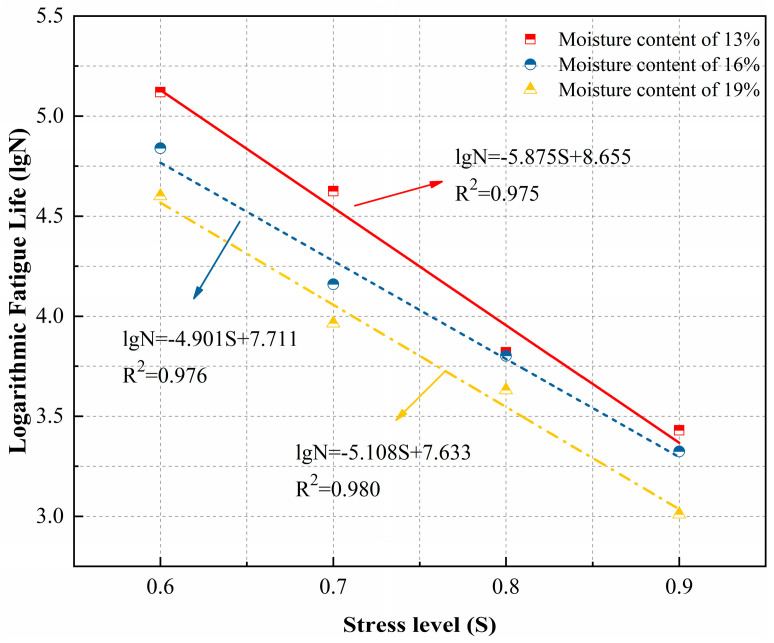
P-S-lgN curve of the Weibull distribution at a 0.95 guarantee rate.

Based on the data in Table 8, a single-logarithmic curve was employed to analyze the functional relationship between the stress levels of loess specimens after polymer grouting and their fatigue life. Curves based on the Weibull distribution were plotted for each guarantee rate and moisture content, as shown in Figure 11 and Figure 12.

Based on the fitting equations from Figure 11 and Figure 12, the single-log fatigue equations for polymer grout-reinforced loess specimens at different moisture contents, with Weibull distribution guarantee rates of 50% and 95%, are presented in Table 9.

## 4. Numerical Simulation Study on Grouting Reinforcement of Loess Subgrades

Fatigue testing provides empirical data to predict the fatigue behavior of materials or structures, yet it is time-consuming and labor-intensive. This is particularly true for large-scale structures such as loess subgrades, where both the complexity and cost of testing significantly increase. Numerical simulation, however, can efficiently replicate stress distributions and fatigue crack propagation under actual operating conditions, thereby predicting structural fatigue life cost-effectively. Consequently, this study integrated the Fe-safe fatigue calculation program with the Abaqus numerical simulation software and version of the software was Abaqus 6.14. First, a detailed three-dimensional numerical model of the road was established using the Abaqus finite element software. Subsequently, the Fe-safe fatigue calculation program was employed to determine the fatigue life of the loess subgrade reinforced with permeable polyurethane polymer grouting. This provides a design reference for the fatigue life assessment of permeable polyurethane polymer grouting technology in practical loess road engineering applications.

### 4.1. Calculate Model Parameters

#### 4.1.1. Model Dimensions and Road Surface Parameter Settings

It is impractical to construct an infinitely large model when conducting finite element analysis, given computational resources and time constraints. Therefore, it is necessary to select a reasonable model size that both reflects actual engineering characteristics and is suitable for finite element numerical simulation analysis using computer software. This study employed a three-dimensional finite element model with dimensions of 3 m × 3 m × 3.26 m. The pavement structure comprised five layers: asphalt surface course, asphalt intermediate course, asphalt base course, semi-rigid base course, and sub-base course. As the primary focus of this research was the subgrade, empirical values were adopted for the pavement structure parameters. The parameters for each pavement layer are detailed in Table 10.

#### 4.1.2. Subgrade Parameter Settings

This section focuses on the study of subgrade fatigue life. The Mohr–Coulomb model accounts for the isotropic hardening and softening of materials and is widely applied in the design and calculation of road engineering; hence, the subgrade was modeled using the Mohr–Coulomb model. Key parameters such as the subgrade’s elastic modulus, cohesion, and internal friction angle were required to accurately simulate the stress state of the subgrade before and after grouting reinforcement. The elastic modulus post-grouting reinforcement was obtained using compressive strength tests, while the cohesion and internal friction angle were determined via direct shear tests. Considering that Poisson’s ratio exerted a relatively minor influence on the subgrade compared with other parameters, an empirical value was employed for this ratio.

**Figure 13:**
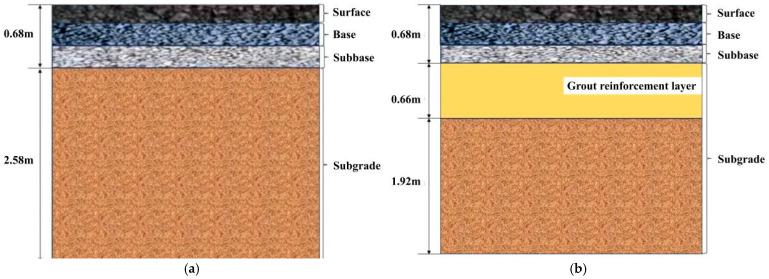
Schematic diagram of road structure before and after grouting reinforcement. (**a**) Schematic diagram of road structure before grouting reinforcement; (**b**) Schematic diagram of road structure following grouting reinforcement.

The research findings indicate that the loess specimens exhibited optimal mechanical properties following grouting reinforcement when the moisture content was 13%. Building on this foundation, this section will focus on investigating the fatigue life of the subgrade material before and after grouting reinforcement at a moisture content of 13%. For the subgrade before grouting reinforcement, parameter settings adhered to the standard parameters for a fully compacted subgrade. Considering the diffusion characteristics and preliminary test results, the thickness of the polymer grout reinforcement layer was set at 66 cm. The relevant subgrade soil parameters are presented in Table 11, while schematic diagrams of the road model before and after grouting reinforcement are shown in Figure 13.

#### 4.1.3. Loads and Boundary Conditions

**Figure 14:**
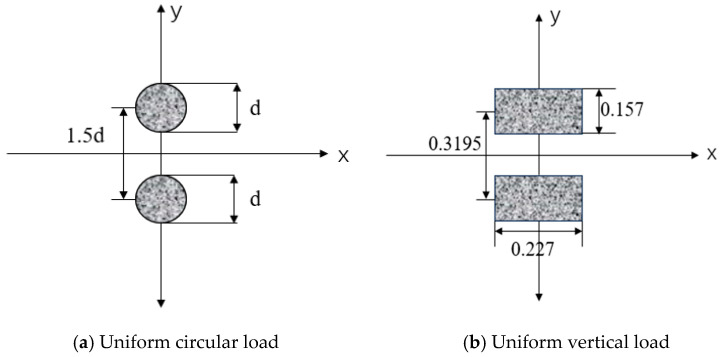
Load application method.

**Figure 15:**
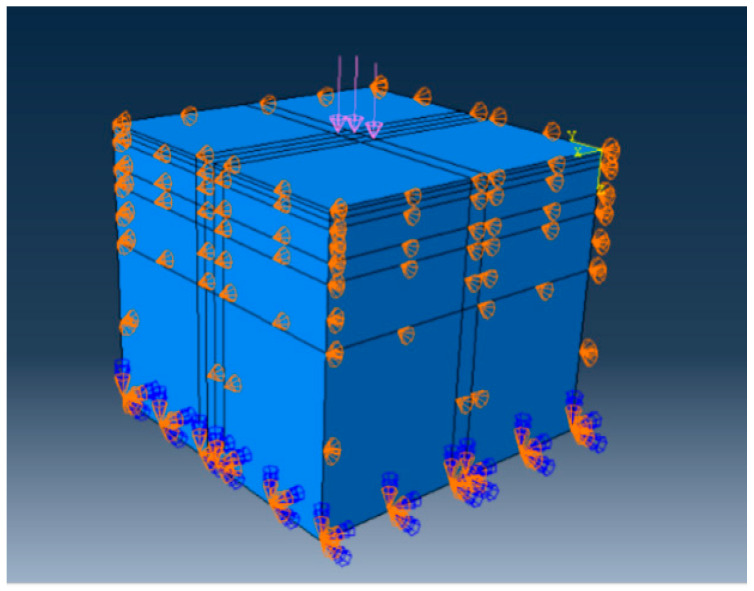
Road model.

In the road model, the grid cell type employed C3D8R cells, with the *X*-axis perpendicular to the direction of vehicular loading, the *Y*-axis parallel to the direction of vehicular loading, and the *Z*-axis extending along the subgrade depth. The boundary conditions were set as follows: displacement in the X- and Y-directions was constrained along the vertical and parallel vehicular loading directions, respectively; displacement in the X-, Y-, and Z-directions was constrained at the subgrade base. In this model, it was assumed that all road structural layers were fully continuous and exhibited no sliding friction; consequently, the contact mode for each layer was set to ‘fully bonded’. A standard vehicle load was applied to the pavement surface, with a vertical pressure of 0.7 MPa. This study converted the double-circle uniformly distributed vertical load (as shown in Figure 14) into an equivalent rectangular uniformly distributed vertical load (as shown in Figure 15). This loading method simplified calculations and more closely approximated real-world conditions. The tire contact patch width and length were 0.157 m × 0.227 m. The established road model is depicted in Figure 15.

### 4.2. Analysis Based on Abaqus Computational Results

**Figure 16:**
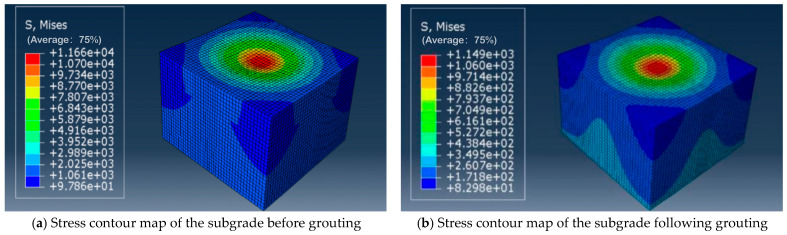
Stress contour map of subgrade.

**Figure 17:**
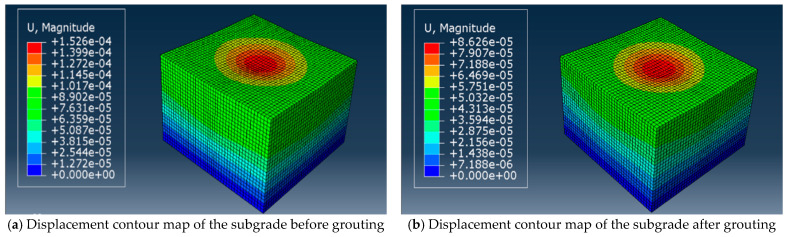
Cloud maps of subgrade displacement.

This study addressed solely the fatigue life of the subgrade; hence, the Abaqus results presented herein pertain exclusively to the subgrade section. Stress and displacement contour plots for the subgrade before and after grouting are shown in Figure 16 and Figure 17, respectively, with stress units in Pa and displacement units in meters. Both the maximum stress point and the maximum displacement point occurred at the subgrade center directly beneath the wheel load. The maximum stress at the top of the subgrade in the non-grouting group was 1.166 × 10^4^ Pa, while that in the grouting group was 1.149 × 10^3^ Pa. Following grouting, the maximum stress at the subgrade top decreased by nearly a factor of ten. The maximum displacement at the subgrade top before grouting was 1.526 × 10^−1^ mm. Following grout reinforcement, the displacement at the subgrade top was 8.626 × 10^−2^ mm, with subgrade settlement also reduced compared with the non-grouted condition. This is attributable to the grout reinforcement layer effectively dispersing and transmitting loads, thereby mitigating subgrade deformation.

The compressive stress σ = 1.166 × 10^4^ Pa transmitted from the pavement to the top of the subgrade in the non-grouted group was determined according to the Abaqus calculation results. When the vehicle load is simplified as a uniformly distributed rectangular load, the compressive stress at the top of the subgrade can be verified using Equation (13):

(13) $$\sigma_{j}=\frac{blp}{\left(b+2Ztan\theta \right)\left(l+2Ztan\theta \right)}.$$

In the formula, σ_j_ denotes the stress at the top of the subgrade (kPa), b represents the length of the rectangular uniformly distributed load (m), l denotes the width of the rectangular uniformly distributed load (m), P signifies the intensity of the rectangular uniformly distributed load (kPa), z indicates the depth beneath the load application point, i.e., the distance from the subgrade top to the road surface (m), and θ denotes the stress diffusion angle (°).

The length b of the rectangular uniformly distributed load was 0.227 m, the width l was 0.157 m, and the load intensity P was 0.7 MPa. The distance Z from the subgrade crown to the road surface was 0.68 m, with an angle of 30°. Substituting into Equation (13) yielded σ_j_ = 1.272 × 10^4^ Pa. This value is consistent with the Abaqus calculation result σ = 1.166 × 10^4^ Pa, demonstrating the accuracy of the Abaqus model.

### 4.3. Subgrade Fatigue Life Prediction Based on Fe-Safe

In practical engineering applications, subgrades rarely fail under static loading; their primary failure mode is typically fatigue failure induced by cyclic loading. Consequently, research into subgrade fatigue failure is particularly important. Domestic and international scholars have conducted few in-depth investigations into the fatigue behavior of loess subgrades using the fatigue analysis software Fe-safe 6.14. Given that traditional subgrade fatigue testing is both time-consuming and labor-intensive, this study employed the Fe-safe fatigue software to analyze the fatigue life of loess subgrades before and after grouting reinforcement. Initially, the Abaqus software was employed to compute the stress state of the subgrade. Subsequently, the resulting ODB output file was imported into Fe-safe for detailed fatigue life analysis, thereby evaluating the fatigue life of loess subgrades before and after grouting.

#### 4.3.1. Parameter Settings and Operational Procedures

**Figure 18:**
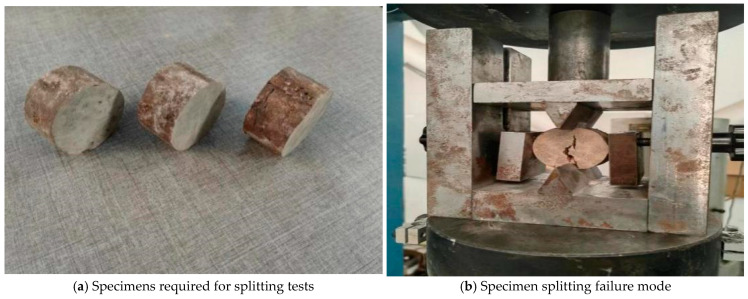
Fatigue test process diagram.

A roadbed’s stress–fatigue curve must be established before analyzing its fatigue life. The Fe-safe software could generate an approximate S-N curve based on the material properties, elastic modulus, and tensile strength. The elastic modulus was derived from Section 2, while the tensile strength was determined via splitting tests [45]. The specimens for fatigue testing were prepared using the specimen preparation method described in Section 2 and, subsequently, cut into the required dimensions using a cutting machine. The specimens comprised cylindrical specimens with a diameter of 50 mm and a height of 20 mm. The cut specimens are shown in Figure 18a. The cut specimens were then placed on the testing machine for testing. The splitting failure mode of the specimen is shown in Figure 18b.

This section focuses solely on the investigation of a single condition where the subgrade moisture content was 13%. Consequently, only the tensile strength of specimens with a 13% moisture content was tested before and after grouting. Three parallel tests were conducted on specimens before and after grouting to ensure accuracy, averaging the tensile strengths obtained from splitting tests. The calculations yielded tensile strengths of 0.19 MPa and 1.72 MPa for the specimens before and after grouting, respectively.

Upon obtaining the required parameters, the ODB results file generated using Abaqus was imported into the Fe-safe software for further processing. After this, all stress states from the analysis steps were imported to perform a full-cycle analysis. The units were defined, ensuring consistency with those used in Abaqus. Pa, N, and m were selected as the units. The components to be analyzed were selected, namely, the grouted subgrade and the entire subgrade. The material properties for the analyzed components were defined, inputting parameters such as tensile strength and elastic modulus. The software automatically generated the material’s stress–strain curve, employing the Goodman model for calculation [46]. The calculation was submitted. The Fe-safe output was an ODB file; importing this ODB results file into Abaqus enabled the subgrade’s fatigue life contour plots to be viewed.

#### 4.3.2. Fatigue Life Prediction for Grouting Reinforcement Layers

**Figure 19:**
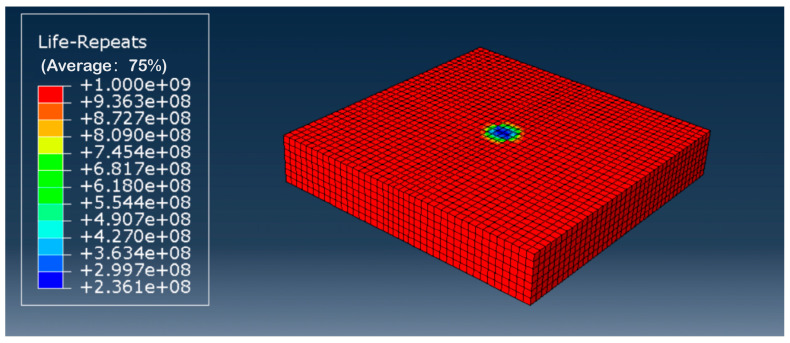
Fatigue life contour map of grouted reinforcement layer in road subgrade.

This section analyzes the fatigue life of the grouted reinforcement layer within the subgrade. The fatigue life contour map for the grouted reinforcement layer is shown in Figure 19. The weakest point in the fatigue life was located directly beneath the wheel load, with a fatigue life of 2.707 × 10^8^ cycles. The fatigue life of the high-injection reinforcement layer was calculated using fatigue equations. Substituting the stress levels at the top of the subgrade into the corresponding fatigue equation yielded its fatigue life. The stress level was calculated using the formula S = σ/fcu, where σ represents the compressive stress at the top of the subgrade, and fcu denotes the compressive strength. Following grouting, the compressive strength fcu of the test specimen under the optimal combination of factors was 6.11 MPa. The numerical simulation results indicate that the stress at the top of the subgrade after grouting was 1.149 × 10^3^ Pa. Therefore, the stress level at the top of the loess subgrade after grouting was S = σ/f_cu_ = 0.001149/6.11 = 0.00024. Substituting the calculated stress level into the corresponding fatigue equation yielded a logarithmic fatigue life, lgN, of 8.48 at a 0.5 assurance level, resulting in N = 3.02 × 10^8^. At a 0.95 assurance level, the logarithmic fatigue life, lgN, equaled 8.65, yielding N = 4.47 × 10^8^. This result is broadly consistent with the fatigue life cycles calculated using the Fe-safe method in this section. Although the specific figures differ, the outcomes from both approaches lie within the same order of magnitude, further validating the reliability of the fatigue equation derived using theoretical analysis.

#### 4.3.3. Prediction of Fatigue Life for Subgrade

**Figure 20:**
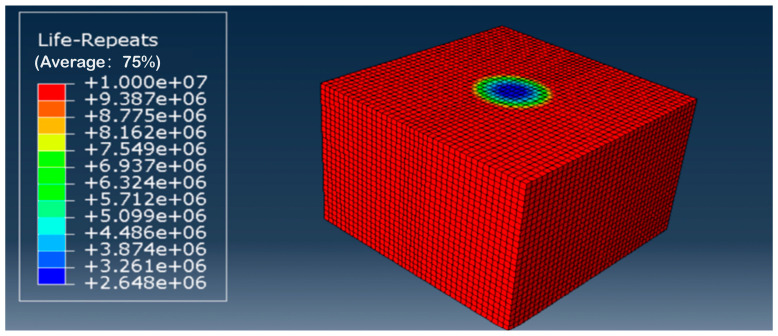
Contour map of subgrade fatigue life before grouting reinforcement.

**Figure 21:**
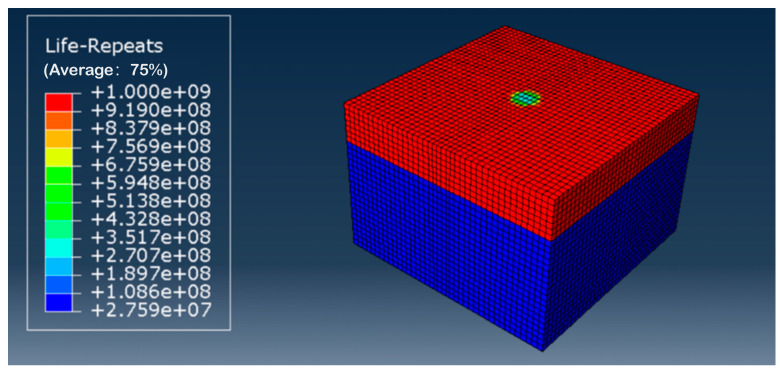
Contour map of fatigue life of roadbed following grouting reinforcement.

The contour plots of the fatigue life of the subgrade before and after grouting reinforcement are shown in Figure 20 and Figure 21, respectively. The fatigue life of the subgrade before grouting was 2.648 × 10^6^ cycles, whereas it increased to 2.759 × 10^7^ cycles after grouting reinforcement, representing a tenfold improvement in fatigue life compared with the pre-grouting state. Even with an injection reinforcement layer thickness of merely 66 cm, the enhancement to the overall subgrade fatigue life remained significant. This is attributable to the protective effect exerted by the injection reinforcement layer upon the underlying subgrade. Further analysis revealed that before grout reinforcement, the most vulnerable zone for fatigue life in the subgrade was located at its crown, directly beneath wheel loads. However, for the reinforced subgrade model, the weakest point for fatigue life appeared in the lower subgrade region, where grout diffusion had failed to reach. This contrasts with the model’s static analysis results, which indicated maximum stress values at the embankment’s top. This discrepancy arises because the tensile strength and elastic modulus of the lower half of the grout-reinforced model were significantly lower than those of the polymer-reinforced layer in the upper half. Although static analysis showed the highest stress values at the model’s top, the inferior mechanical properties of the lower section relative to the reinforced layer resulted in a reduced fatigue life compared with the reinforced portion. This shift in the critical fatigue zone underscores a fundamental design principle for polymer grouting reinforcement: the treatment transforms the failure mode from a surface-initiated fatigue problem to one governed by the properties of the underlying transition zone between the reinforced and native materials. From a design perspective, this observation necessitates a holistic approach where the geometry of the reinforcement zone is optimized not only to reduce surface stress but also to ensure that the resulting stress re-distribution does not merely relocate the critical fatigue damage to an inaccessible depth.

Following the grouting reinforcement of loess subgrades with permeable polyurethane polymers, a composite structure forms between the polymer and soil particles. This enhances the cohesion of the soil mass while significantly improving its resistance to external loads. The composite structure effectively distributes and transmits loads, reducing subgrade deformation and damage. It provides protective benefits to the underlying subgrade, thereby extending its fatigue life and enhancing its load-bearing capacity.

## 5. Conclusions

This study employed indoor compressive fatigue testing to investigate the fatigue performance of loess specimens reinforced with permeable polyurethane polymer grouting, as well as the effects of moisture content and stress levels on specimen fatigue life. A fatigue life equation for permeable polyurethane polymer grouted loess specimens was established based on experimental data. A three-dimensional road model was constructed utilizing the Abaqus numerical simulation software. Combined with the Fe-safe fatigue calculation program, the fatigue life of loess subgrades before and after grouting reinforcement was computed. The enhancement effect of permeable polyurethane polymer grout reinforcement on the fatigue life of loess subgrades was analyzed, yielding the following primary conclusions:

(1) Following grouting reinforcement, loess specimens achieved a fatigue life of 200,000 cycles. Stress levels and the moisture content significantly influenced the fatigue life of the grouted specimens. As stress levels increased, the load borne by the specimens progressively approached their bearing capacity, resulting in reduced fatigue life. At equivalent stress levels, fatigue life decreased with rising moisture content.
(2) The fatigue failure process of the grouted specimens was categorized into three stages based on their respective contributions to the total fatigue life. During the latter phase of the third stage, the specimens exhibited a ‘multiple-crack’ failure pattern. This indicated that the specimens reinforced with permeable polyurethane polymer grouting achieved a more uniform stress distribution, mitigating localized stress concentrations. Consequently, their resistance to fatigue loading was enhanced, thereby extending their fatigue life.
(3) Mathematical statistical methods verified that the fatigue life of the loess specimens after permeable polyurethane polymer grouting followed a two-parameter Weibull distribution, exhibiting a good linear relationship between the logarithmic fatigue life and the stress level. Based on this, a fatigue life prediction equation for grouted loess specimens was established, accounting for different moisture contents and assurance factors.
(4) The fatigue life cycles of grouted loess subgrades calculated using the Fe-safe fatigue program significantly exceeded the pre-grouting values. The polymer grout reinforcement layer achieved a fatigue life of 2.707 × 10^8^ cycles, demonstrating its efficacy in enhancing loess subgrade fatigue resistance.
(5) The fatigue life of the permeable polyurethane polymer grout reinforcement layers predicted using the Fe-safe fatigue calculation software aligned with the results obtained from the experimentally derived fatigue equations at comparable magnitudes. This validates the accuracy of the numerical model while providing a reference basis for predicting the fatigue life of permeable polyurethane polymer grout-reinforced loess subgrades in practical engineering applications.

## Figures and Tables

**Table 1:** Grain size analysis results.

Particle Size (mm)	2	1	0.5	0.25	0.075
Percentage below this particle size (%)	100	98.1	96.5	94.6	79.8

**Table 2:** Basic physical properties of loess.

Soil Particle Proportion	Plastic Limit (%)	Liquid Limit (%)	Plasticity Index	Maximum Dry Density (g/cm^3^)	Optimum Moisture Content (%)
2.70	13.4	31.9	18.7	1.76	12.7

**Table 3:** Physical properties of polymer materials.

Component	Size Viscosity (MPa·s)	Slurry Density (g/cm^3^)	Gelation Time (min)
Mixture A	4	1.05	30
Mixture B	23	1.20	

**Table 4:** The molecular weight of polymer materials.

Ingredient Name		Molecular Formula	Content
A	Polyether polyols	C_8_H_22_O_7_	30–60%
	Tris Nphosphate	C_9_H_18_C_l3_O_4_P	10–40%
B	Polymeric MDI	C_15_H_10_N_2_O_2_	50–70%
	MDI	C_15_H_10_N_2_O_2_	30–50%

**Table 5:** Peak load stress applied at each stress level and moisture content.

Moisture Content (%)	Load Applied at Various Stress Levels (MPa)			
	0.6 Stress Level	0.7 Stress Level	0.8 Stress Level	0.9 Stress Level
13	3.6	4.3	4.9	5.5
16	3.1	3.6	4.1	4.6
19	2.4	2.8	3.2	3.6

**Table 6:** The results of fatigue life.

Moisture Content (%)	Fatigue Life of Specimens Under Different Stress Levels (Number of Cycles)			
	0.6	0.7	0.8	0.9
13	200,005	105,879	34,061	6114
	185,806	96,748	26,788	5047
	169,607	75,699	20,398	4612
	160,927	66,762	13,206	3849
16	156,764	76,501	20,322	4369
	148,748	58,167	18,000	4057
	12,661	40,329	14,218	3581
	99,541	30,663	10,459	2870
19	90,567	48,529	9647	2846
	82,671	39,668	8867	2347
	71,185	29,226	7349	1897
	56,507	18,489	7012	1630

**Table 7:** Logarithmic fatigue life of specimens with different moisture contents and stress levels.

Moisture Content (%)	Specimen Number	Logarithmic Fatigue Life of Specimens at Different Stress Levels (lgN)				Guarantee Rate P = 1 − i/(*n* + 1)	−ln ln(1/P)
		0.6	0.7	0.8	0.9		
13	1	5.2066	4.8245	4.1208	3.5853	0.8	1.5
	2	5.2294	4.8791	4.3096	3.6639	0.6	0.6717
	3	5.2691	4.9856	4.4279	3.703	0.4	0.0874
	4	5.301	5.0248	4.5323	3.7863	0.2	−0.4759
16	1	4.998	4.4866	4.0195	3.4579	0.8	1.5
	2	5.0816	4.6056	4.1528	3.554	0.6	0.6717
	3	5.1725	4.7647	4.2553	3.6082	0.4	0.0874
	4	5.1952	4.8837	4.3078	3.6404	0.2	−0.4759
19	1	4.7521	4.2669	3.8014	3.2122	0.8	1.5
	2	4.8524	4.476	3.9263	3.2781	0.6	0.6717
	3	4.9174	4.5984	3.9799	3.3705	0.4	0.0874
	4	4.957	4.686	4.0402	3.4542	0.2	−0.4759

**Table 8:** Logarithmic fatigue life of specimens at different stress levels and moisture contents.

Moisture Content (%)	Guarantee Rate	−ln ln(1/P)	Logarithmic Fatigue Life of Specimens at Different Stress Levels (lgN)			
			0.6	0.7	0.8	0.9
13	0.50	0.3665	5.23	4.92	4.36	3.69
	0.95	2.9702	5.12	4.63	3.82	3.43
16	0.50	0.3665	5.12	4.70	4.20	3.57
	0.95	2.9702	4.84	4.16	3.80	3.32
19	0.50	0.3665	4.88	4.53	3.95	3.34
	0.95	2.9702	4.60	3.97	3.63	3.01

**Table 9:** Fatigue equations for specimens at different moisture contents and guarantee rates.

Moisture Content (%)	Fatigue Equations at Different Safety Factors	
	0.50 Guarantee Rate	0.95 Guarantee Rate
13	lg *n* = −5.24 S + 8.49	lg *n* = −5.88 S + 8.66
16	lg *n* = −5.15 S + 8.26	lg *n* = −4.90 S + 7.71
19	lg *n* = −5.20 S + 8.08	lg *n* = −5.11 S + 7.63

**Table 10:** Structural layer parameters of pavement.

Structure Layer	Thickness (cm)	Density (kg/cm^3^)	Modulus of Elasticity (MPa)	Poisson’s Ratio
Asphalt surface layer	4	2300	1300	0.30
Asphalt middle layer	6	2300	1300	0.30
Asphalt base course	8	2300	1300	0.30
Semi-rigid base layer	30	2000	1600	0.25
Sub-base	20	2000	1200	0.25

**Table 11:** Parameters of subgrade soil material.

Material Name	Modulus of Elasticity (MPa)	Cohesion (kPa)	Internal Friction Angle (°)	Poisson’s Ratio
Subgrade after grouting reinforcement	122.3	258	45.8	0.3
Subgrade before grouting reinforcement	40	12	25	0.25

## Data Availability

The original contributions presented in this study are included in the article. Further inquiries can be directed to the corresponding author.
